# Hemorrhagic Brain Tumor As the Initial Presentation of Metastatic Testicular Yolk Sac Tumor: A Case Report and Review of the Literature

**DOI:** 10.7759/cureus.109078

**Published:** 2026-05-18

**Authors:** Badr E Hafiz, Fahad M Okal, Abdulaziz Hamzah, Abdelrazak Meliti, Mohammed Homoud

**Affiliations:** 1 Department of Neurosciences, King Faisal Specialist Hospital and Research Centre, Jeddah, SAU; 2 Department of Surgery, King Abdulaziz Medical City, Ministry of National Guard Health Affairs, Jeddah, SAU; 3 Department of Pathology and Laboratory Medicine, Alfaisal University, Riyadh, SAU; 4 Department of Anatomic Pathology, King Faisal Specialist Hospital and Research Centre, Jeddah, SAU

**Keywords:** brain metastasis, craniotomy, germ cell tumor, hemorrhagic metastasis, yolk sac tumor

## Abstract

Yolk sac tumors (YSTs) are highly malignant germ cell neoplasms, most commonly arising in the gonads. Intracranial involvement is exceptionally rare and typically affects the pineal or suprasellar regions. Metastatic YST presenting as a hemorrhagic brain lesion is an exceedingly unusual occurrence that poses significant diagnostic and therapeutic challenges. We report the case of a 26-year-old male who presented with rapidly progressive left-sided weakness and headache. Imaging revealed a right frontoparietal hemorrhagic mass with marked vasogenic edema and midline shift. The patient underwent urgent right frontal craniotomy and near-total tumor excision via a transsulcal approach. Postoperatively, neurological recovery was rapid, with near-complete motor improvement within days. Systemic evaluation revealed multiple pulmonary and para-aortic metastases and an enlarged right testis. Serum α-fetoprotein (AFP) was markedly elevated, and histopathological examination confirmed the diagnosis of YST. The patient received adjuvant chemotherapy, followed by radical orchidectomy and lymph node dissection, achieving normalization of AFP and full functional recovery at one year of follow-up. This case underscores the importance of considering germ cell tumors in the differential diagnosis of hemorrhagic brain lesions, particularly in young males. Early surgical intervention, combined with systemic chemotherapy, can lead to favorable neurological and oncologic outcomes even in such rare and aggressive presentations.

## Introduction

Yolk sac tumors (YSTs), also referred to as endodermal sinus tumors, are malignant germ cell neoplasms that form a distinct histological subtype within the spectrum of non-seminomatous germ cell tumors (NSGCTs) [1]. These tumors develop from primordial germ cells, which are the embryonic precursors of gametes such as sperm and oocytes [1,2]. During early embryogenesis, these pluripotent cells arise in the wall of the yolk sac near the allantois and migrate along the midline toward the developing gonadal ridges [2]. In some cases, germ cells may deviate from their normal migratory path or become trapped along midline structures, including the mediastinum, retroperitoneum, sacrococcygeal area, or intracranial regions such as the pineal gland. When such misplaced germ cells undergo malignant transformation outside the gonads, they can form extragonadal germ cell tumors, including YSTs [2].

The frequency of central nervous system (CNS) metastases in YST is relatively low, occurring in about 1-3% of patients with advanced germ cell tumors [3]. Mixed tumor histology or extensive pulmonary metastases may provide a pathway for hematogenous dissemination to the brain [3]. In early-stage disease, CNS involvement is exceedingly uncommon, with an incidence below 1%, particularly in cases initially presenting as hemorrhagic brain metastasis from YST [4].

Histologically, YSTs display diverse architectural patterns, most commonly the reticular (microcystic) and solid forms. However, the defining microscopic feature is the Schiller-Duval body - a structure characterized by a central vessel surrounded by tumor cells within a cystic space lined by flattened tumor cells - which is considered pathognomonic for YST [5].

This case is important to report because hemorrhagic brain metastasis as the initial manifestation of metastatic testicular YST is exceedingly rare and may mimic other more common intracranial hemorrhagic lesions. Reporting this case highlights the diagnostic challenges, emphasizes the importance of systemic and testicular evaluation in young males presenting with hemorrhagic brain tumors, and demonstrates the favorable neurological and oncological outcomes that can be achieved with prompt multidisciplinary management.

## Case presentation

In March 2025, a previously healthy 26-year-old male presented to the emergency department with a two-day history of rapidly progressive left-sided weakness associated with intermittent moderate headaches. Neurological examination revealed left-sided weakness with Medical Research Council (MRC) grade 3/5 power in both the upper and lower limbs, with intact sensation. The remainder of the neurological examination was unremarkable.

Given the acute neurological deterioration and significant mass effect on neuroimaging, the initial clinical priority was urgent neurosurgical assessment and stabilization. Therefore, although a general physical examination was performed at presentation, a detailed systemic and focused testicular examination was not fully completed or documented before emergency surgical intervention.

An urgent non-contrast computed tomography (CT) scan of the brain (Figure 1) demonstrated a large heterogeneously hyperdense intraparenchymal lesion centered in the right centrum semiovale involving the precentral and postcentral gyri, measuring 4.5 × 4.1 × 4 cm, with internal hemorrhagic components, marked surrounding vasogenic edema, and approximately 6.6 mm midline shift.

**Figure 1:**
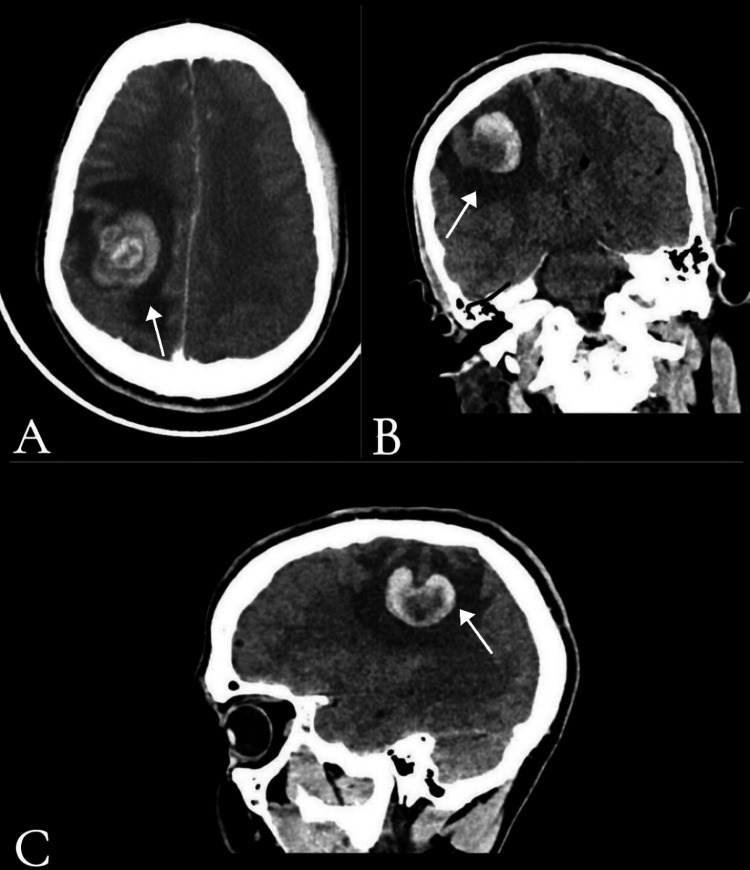
Preoperative CT of the brain without contrast (A) Axial, (B) coronal, and (C) sagittal images showing a large heterogeneously hyperdense intraparenchymal lesion (white arrows) at right centrum semiovale with involvement of precentral and postcentral gyrus measuring 4.5 x 4.1 x 4 cm in anteroposterior (AP), transverse, and craniocaudal (CC) dimensions, respectively. It has a significant surrounding vasogenic edema and midline shift of 6.6 mm.

Subsequent magnetic resonance imaging (MRI) of the brain (Figure 2) demonstrated a heterogeneously enhancing hemorrhagic intraparenchymal lesion centered in the right centrum semiovale involving the precentral and postcentral gyri, appearing predominantly iso- to hypointense on T1-weighted imaging with heterogeneous internal signal characteristics consistent with blood products. Extensive surrounding T2-weighted/fluid-attenuated inversion recovery (T2/FLAIR) hyperintense vasogenic edema and significant mass effect were also noted.

**Figure 2:**
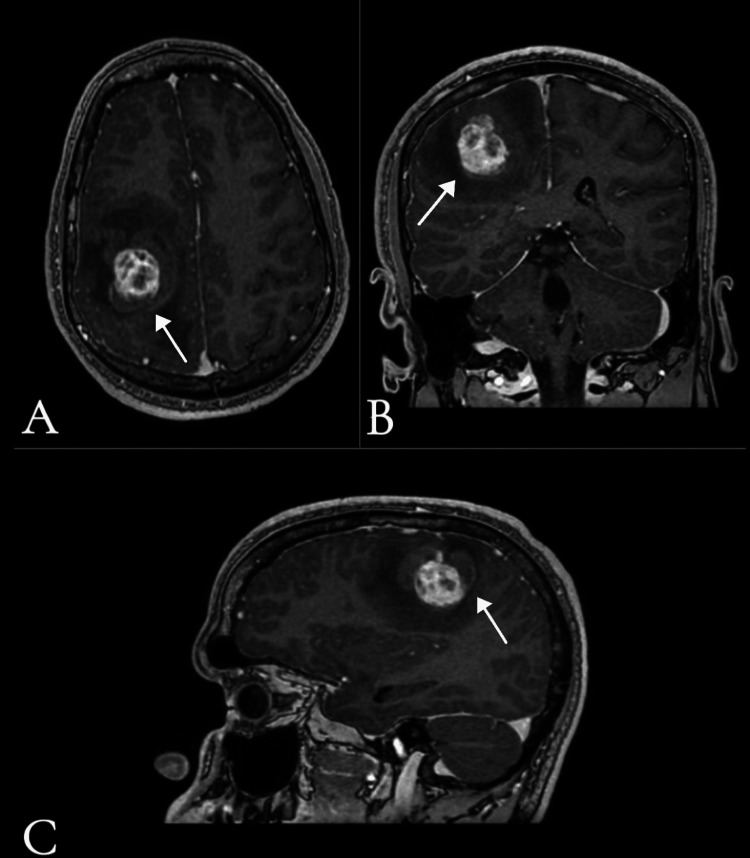
Preoperative MRI of the brain T1 with contrast (A) axial, (B) coronal, and (C) sagittal images showing a large hyperintense intraparenchymal lesion (white arrows) with heterogeneous contrast enhancement noted at right centrum semiovale with involvement of precentral and postcentral gyrus measuring 4.5 x 4.1 x 4 cm in anteroposterior (AP), transverse, and craniocaudal (CC) dimensions, respectively. It has a diffuse surrounding vasogenic edema and midline shift of 6.6 mm.

Due to rapid neurological deterioration and radiological evidence of significant mass effect, the patient underwent urgent right frontal craniotomy on the day of admission (day 1). A transsulcal approach was performed along the central sulcus until the tumor surface was exposed. Gentle circumferential dissection of the tumor from the surrounding white matter was carried out under microscopic visualization. Abnormal vessels were coagulated, and stepwise separation of the lesion from the surrounding brain parenchyma was carefully achieved. The tumor was excised in a piecemeal fashion, and its membrane was meticulously removed. Intraoperative fresh frozen section analysis of the resected lesion demonstrated a non-glial malignant neoplasm suspicious for metastatic disease. The patient was extubated in the operating theater and transferred to the surgical intensive care unit for 24-hour observation.

On postoperative day 1, the patient demonstrated early neurological improvement with recovery of motor strength from 3/5 to -4/5 MRC grade in both the left upper and lower limbs. Postoperative CT brain (Figure 3) demonstrated satisfactory resection of the intraparenchymal lesion with expected postoperative changes. Based on the preliminary pathological findings in the intraoperative fresh frozen section, further systemic evaluation was pursued to identify the primary tumor source. A comprehensive systemic examination, including focused testicular examination, was performed and revealed asymmetric enlargement of the right testis. Subsequent scrotal ultrasonography demonstrated a large, heterogeneous, ill-defined right testicular mass suspicious for malignancy.

**Figure 3:**
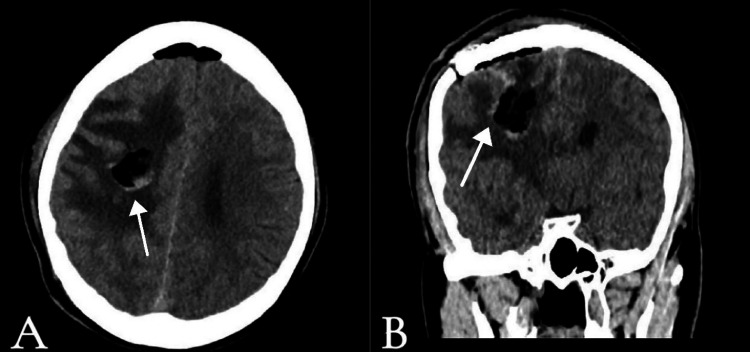
Postoperative CT of the brain without contrast (A) Axial and (B) coronal images showing postoperative changes in the form of surgical site of pneumocephalus (white arrows) extending into both frontal regions, hemorrhagic components noted at the surgical site, and white matter of the right frontal parietal region measuring approximately 1.6 x 0.7 cm. Focal area of fat density noted adjacent to the intraparenchymal hemorrhage, likely surgical material.

On postoperative day 2, CT of the chest, abdomen, and pelvis demonstrated multiple pulmonary metastatic lesions, necrotic para-aortic lymphadenopathy, and asymmetric enlargement of the right testis. Routine laboratory investigations and serum tumor markers (Table 1) revealed markedly elevated alpha-fetoprotein (AFP) levels with normal beta-human chorionic gonadotropin (β-hCG).

**Table 1:** Routine labs and tumor markers

Parameter	Result	Reference Range
Alpha-fetoprotein (AFP)	14,000 IU/mL	<5.8 IU/mL
Beta-human chorionic gonadotropin (β-hCG)	3	<5 IU/L
Hemoglobin (Hb)	14.5 g/dL	13.0-17.0 g/dL
White blood cells (WBC)	7.2×10^9^/L	4.0-11.0 ×10^9^/L
Platelets	374×10^9^/L	150-400 ×10^9^/L
Creatinine	0.8 mg/dL	0.7-1.3 mg/dL
Alanine aminotransferase (ALT)	36 U/L	7-56 U/L
Aspartate aminotransferase (AST)	29 U/L	10-40 U/L

On postoperative day 3, the patient was able to walk with minimal assistance. Neurological examination demonstrated continued improvement in left-sided motor function with 4/5 MRC power grade.

Final histopathological and immunohistochemical analyses confirmed metastatic YST (Figure 4 and Table 2). The case was subsequently discussed at a multidisciplinary tumor board meeting. Given the histopathological findings and elevated tumor markers, the treatment plan included systemic chemotherapy followed by right radical orchidectomy and lymph node dissection.

**Figure 4:**
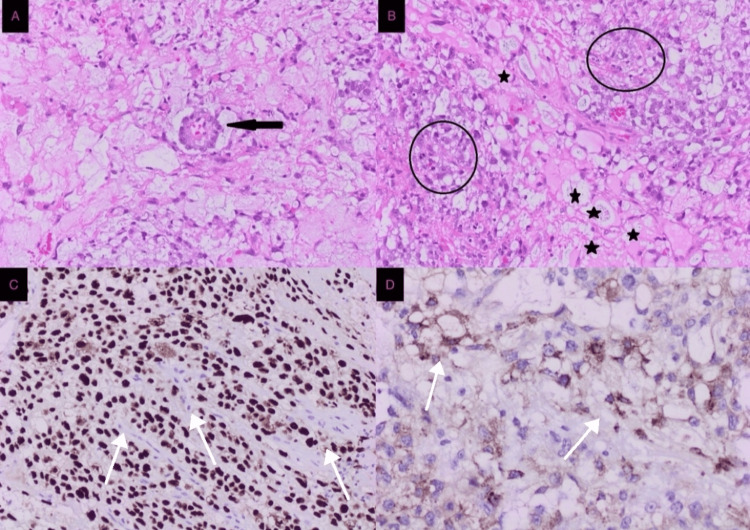
Histopathological analysis of the tumor (A) Hematoxylin and eosin (H&E) section (100x) demonstrates Schiller-Duval body (black arrow). (B) H&E-stained section (100x) shows microcystic pattern (asterisk) and solid pattern (black circles). Immunohistochemistry against SALL4 (white arrows) (C) and glypican-3 (white arrows) (D), at 100x.

**Table 2:** Immunohistochemistry expression profile SALL4: sal-like protein 4, GFAP: glial fibrillary acidic protein, CK8/18: cytokeratin 8/18, OCT4: octamer-binding transcription factor 4, CAM 5.2: cytokeratin (low molecular weight cytokeratin antibody clone CAM 5.2), CD30: cluster of differentiation 30, AE1/AE3: cytokeratin AE1/AE3 (broad-spectrum cytokeratin antibody cocktail), TTF-1: thyroid transcription factor-1, GATA-3: GATA-binding protein 3, Glypican 3: Glypican-3 (heparan sulfate proteoglycan), CD117: cluster of differentiation 117 (c-KIT), Ki-67: Ki-67 proliferation index (marker of cellular proliferation)

	Positive	Negative
1	SALL4	GFAP
2	CK8/18	OCT4
3	CAM 5.2	CD30
4	AE1/AE3	TTF-1
5	GATA-3	-
6	Glypican 3	-
7	CD117	-
8	Ki67: 95%	-

The patient completed five cycles of chemotherapy with significant clinical and biochemical response, including normalization of AFP levels. He subsequently underwent right radical orchidectomy, and histopathological examination confirmed testicular YST. Two months later, the patient underwent pelvic radical lymph node dissection.

Follow-up MRI of the brain eight months after surgery was done (Figure 5) and showed complete resection with no suspected residual/recurrence. On follow-up, one year after surgery, the patient's functional status markedly improved as he can walk independently, and his left-sided weakness completely recovered.

**Figure 5:**
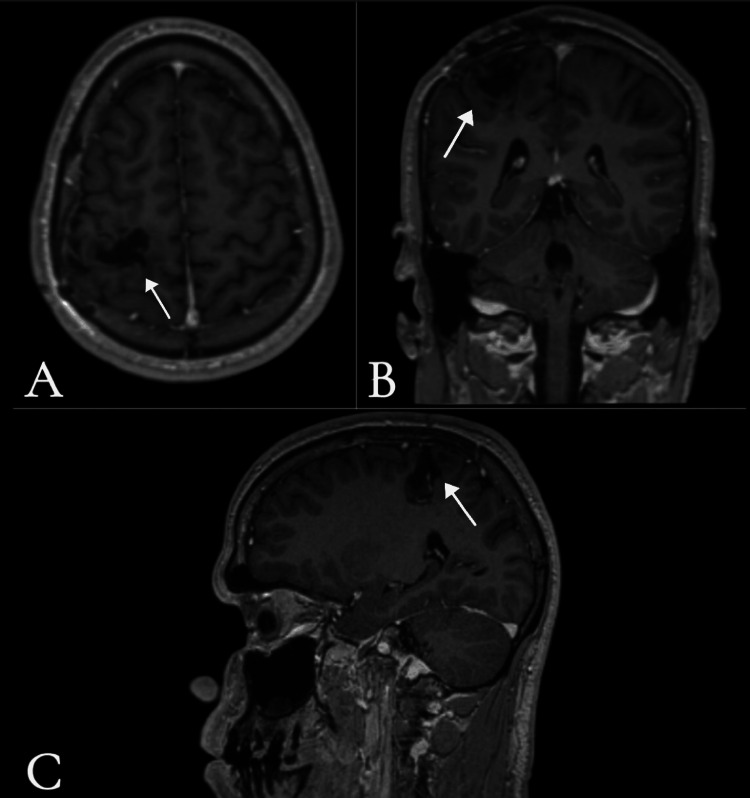
Postoperative MRI of the brain T1 with contrast (A) axial, (B) coronal, and (C) sagittal images showing postsurgical cavity (white arrows) in the right frontal lobe is again noted, currently measuring approximately 2.7 × 2.9 cm, unchanged in size compared to the prior postoperative exam. No new or residual enhancing lesion in the right insular region, confirming previously noted postoperative resolution.

## Discussion

YSTs are uncommon and highly aggressive neoplasms, most frequently identified as masses in the testes or ovaries. Extragonadal germ cell tumors represent only about 2-5% of all germ cell tumors, with common sites including the mediastinum, retroperitoneum, and pineal gland [6]. Intracranial occurrences are extremely rare, typically reported in the pineal and suprasellar regions [6]. Instances of metastatic YST manifesting as intracranial hemorrhage are exceedingly unusual [6].

When evaluating hemorrhagic intracranial masses, the differential diagnosis often includes high-grade gliomas, metastases from other primary tumors (e.g., melanoma, renal cell carcinoma), and vascular malformations [7]. Germ cell tumors are seldom considered, particularly in young adults with acute neurological deterioration [7]. In the present case, clinical findings such as testicular enlargement, para-aortic lymphadenopathy, and elevated tumor markers were crucial in establishing the correct diagnosis.

The exact pathophysiology underlying hemorrhage in YST is not fully elucidated. One proposed mechanism is that rapid tumor proliferation exceeds its vascular supply, resulting in necrosis and subsequent bleeding. Another hypothesis is that highly vascularized tumors may infiltrate and compromise adjacent blood vessels, increasing the risk of hemorrhage [8]. In this patient, the lesion was situated near the central sulcus, which correlates with the observed rapid onset of left-sided hemiplegia.

A literature review was performed using PubMed and Google Scholar databases for articles published between 1980 and 2025 using the keywords "yolk sac tumor," "brain metastasis," "intracranial hemorrhage," and "germ cell tumor." Only English-language case reports and case series describing intracranial YSTs or metastatic YSTs with brain involvement were included (Table 3) [8-20].

**Table 3:** Review of literature for YST cases with brain metastasis PEB: cisplatin, etoposide, bleomycin, PVB: cisplatin, vinblastine, bleomycin, YST: yolk sac tumor

Case No.	First Author and Year	Age/Sex	Tumor Location	Treatment	Outcomes
1	Nakagawa Y, 1980 [8]	Seven-year-old boy	Fourth ventricle	Total resection	Stable at six months
2	Arita N, 1980 [9]	13-year-old girl	Pineal region	Total resection + chemotherapy	Stable at six months
3	Takeda Z, 1985 [10]	12-year-old boy	Right cerebellar hemisphere	Total resection	Stable at six months
4	Kirkove CS, 1991 [11]	25-year-old woman	Pineal region	Total resection + chemotherapy	Stable at 12 months
5	Tsukamoto H, 1992 [12]	14-year-old girl	Cerebellar vermis	Total resection + chemotherapy	Stable at 12 months
6	Takano S, 1993 [13]	38-year-old man	Right parietal lobe (metastasis from testicular YST)	Orchiectomy; craniotomy; chemotherapy (PEB/PVB)	Died six months after orchiectomy
7	Fujiwara T, 1994 [14]	16-year-old boy	Right cerebellar hemisphere	Total resection + chemotherapy	Stable at six months
8	Nakase H, 1994 [15]	Four-year-old boy	Right cerebellar hemisphere	Total resection + chemotherapy	Stable at 12 months
9	Cheon HC, 2006 [16]	Nine-year-old boy	Right cerebellar hemisphere	Subtotal resection + chemotherapy	Stable at six months
10	Kuang H, 2014 [17]	10-year-old boy	Right cerebellar hemisphere	Total resection + chemotherapy	Stable at 12 months
11	Shenoy AS, 2014 [18]	10-year-old boy	Cerebellar vermis	Total resection + chemotherapy	Stable at six months
12	Wu N, 2021 [19]	Six-year-old boy	Cerebellar hemisphere	Subtotal resection; chemotherapy	No recurrence at three months
13	Liu Q, 2024 [20]	22-year-old male	Right basal ganglia - corona radiata	Resection + BEP chemotherapy (discontinued) + whole-brain radiotherapy	Stable at six months with mild cognitive impairment

Analysis of the reported cases demonstrates that intracranial YSTs predominantly occur in pediatric patients and are most frequently located in the posterior fossa or pineal region [8-10,14-19]. Metastatic intracranial involvement from testicular YSTs in adults is exceedingly rare, with only a limited number of reported cases [11,13,20]. Furthermore, most previously reported intracranial YSTs presented with progressive neurological symptoms rather than acute hemorrhage [21]. The present case is therefore unique because the patient presented with sudden neurological deterioration secondary to hemorrhagic metastatic disease involving the motor cortex. Early surgical decompression utilizing a transsulcal approach allowed us to achieve maximal tumor excision with minimal disruption of the surrounding motor cortex, combined with systemic chemotherapy, resulting in an excellent functional outcome. Postoperative recovery was notable for rapid improvement in motor function, observable within six hours, highlighting the potential for favorable neurological outcomes in hemorrhagic brain tumors when timely intervention is undertaken.

Advances in chemotherapy have significantly improved the prognosis of patients with YST [22]. The patient received adjuvant chemotherapy following surgery, and given both the rapid neurological recovery and the tumor’s responsiveness to systemic treatment, a favorable long-term outcome is anticipated.

## Conclusions

Hemorrhagic brain metastasis may rarely represent the initial presentation of metastatic testicular YST in young adults. In patients presenting with acute hemorrhagic intracranial lesions, especially in young males, metastatic germ cell tumors should be considered in the differential diagnosis. Prompt surgical decompression followed by multidisciplinary oncological management can result in excellent neurological recovery and favorable oncological outcomes despite the aggressive nature of the disease.
